# Ocular Adverse Effects of Gefitinib: A Case Report

**DOI:** 10.7759/cureus.29600

**Published:** 2022-09-26

**Authors:** Sandhya Jeria, Archana R Thool, Sachin Daigavane, Samyak Ganjre

**Affiliations:** 1 Department of Ophthalmology, Jawaharlal Nehru Medical College, Datta Meghe Institute of Medical Sciences, Wardha, IND; 2 Department of Dermatology, Jawaharlal Nehru Medical College, Datta Meghe Institute of Medical Sciences, Wardha, IND

**Keywords:** epidermal growth factor receptor, punctate epithelial erosions, non-small cell lung carcinoma, gefitinib, corneal thinning

## Abstract

Gefitinib is a selective epidermal growth factor receptor (EGFR) tyrosine kinase inhibitor. It is used for treating locally advanced or metastatic non-small cell lung carcinoma and is well tolerated systemically. However, sight-threatening ocular adverse effects, like corneal ulcer and perforation, can occur due to the expression of EGFR on limbal and conjunctival epithelia. In this report, we describe a case of a 36-year-old female who presented with loss of eyebrow hair and eyelashes of both eyes and blurring of vision in the right eye. On ocular examination, the patient had anterior blepharitis, madarosis, punctuate epithelial erosions and reduced corneal sensation in both eyes, and corneal thinning in the right eye. On specular microscopy, there was decreased central corneal thickness in both eyes. Treatment with topical antibiotics and lubricating drops led to the resolution of blepharitis and punctate epithelial erosions. This case report aims to create awareness among ophthalmologists and oncologists about the early detection of gefitinib-related ocular adverse effects and timely intervention in patients.

## Introduction

Gefitinib is an orally active selective epidermal growth factor receptor (EGFR) tyrosine kinase inhibitor for treating locally advanced or metastatic non-small cell lung cancer with confirmed EGFR tyrosine kinase mutation [1]. Although gefitinib is used against anomalies in cancer cells, it is associated with certain ocular side effects due to EGFR expression in the eye and its adnexa [2]. In this report, we aim to highlight the ocular adverse effects of gefitinib and its management.

## Case presentation

**Figure 1:**
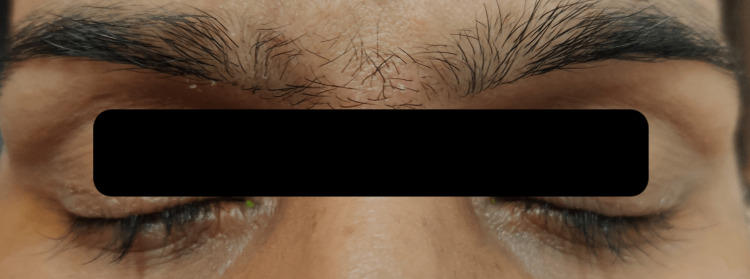
Showing madarosis of eyebrows and eyelashes, and acne between eyebrows

A 36-year-old female presented to the hospital with a history of loss of eyelashes and eyebrow hair in both eyes and blurring of vision in the right eye for the past month. There was associated itching over the eyelid skin and eyebrows. In the right eye, blurring of vision had been gradual in onset and progressive in nature. No history of associated pain, redness, discharge, colored halos, or discomfort with light or ocular trauma was present. The patient had been diagnosed with non-small cell adenocarcinoma of the lung two years ago. Real-time polymerase chain reaction (RT-PCR) had been suggestive of EGFR exon-19 mutation in the tumor. She had been started on gefitinib tablet 250 mg once daily at the time of diagnosis. In addition, she had received nine cycles of chemotherapy with injections of pemetrexed and carboplatin. On general examination, the patient had healed acneiform scars all over the body. On ocular examination, unaided visual acuity was 6/9p in the right eye and 6/6 in the left eye. The best-corrected visual acuity of the right eye was 6/6p with a 0.5 diopter concave lens and that of the left eye was 6/6. Near vision was N/6 and color vision was normal. Acneiform eruptions were present between the eyebrows. Madarosis was present on the medial one-third of both eyebrows. Xerosis was present over both eyelids (Figure 1).

**Figure 2:**
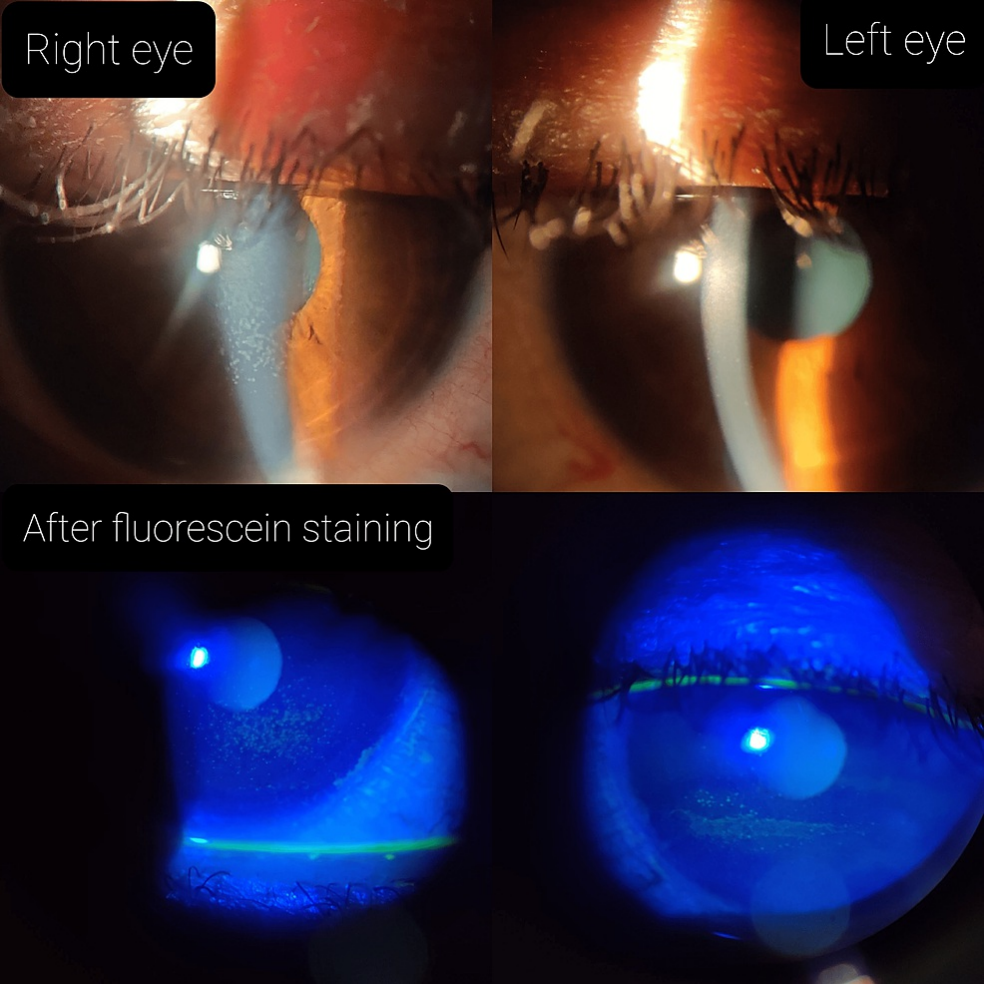
Showing fluorescein stain-positive punctate corneal erosions on the cornea in both eyes

Slit-lamp examination revealed scales and debris along the eyelashes suggestive of anterior blepharitis. There was no conjunctival congestion. Punctate epithelial erosions were present in both eyes, which stained positive with fluorescein (Figure 2).

**Figure 3:**
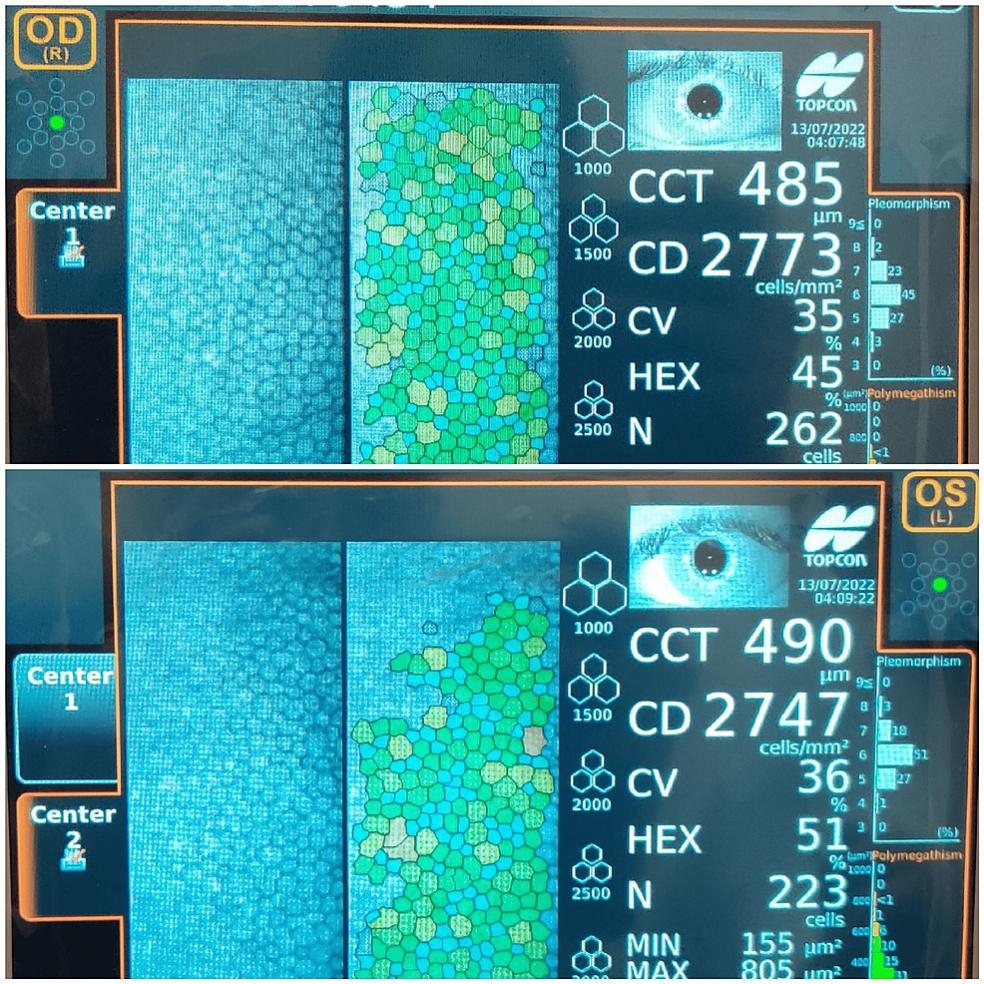
Showing decreased central corneal thickness in both eyes on specular microscopy

There was corneal thinning inferiorly in the right eye. Corneal sensation was elicited using cotton wisp and was found to be decreased in both eyes. The anterior chamber, iris, pupil, lens, and fundus were normal in both eyes. Tear film break-up time in the right eye was nine seconds and it was 10 seconds in the left eye. Schirmer’s I test revealed normal tear production. Specular microscopy revealed a central corneal thickness of 485 microns in the right eye and 490 microns in the left eye. Endothelial cell count on specular microscopy in the right eye was 2773 cells/mm^2^ and it was 2747 cells/mm^2^ in the left eye (Figure 3).

**Figure 4:**
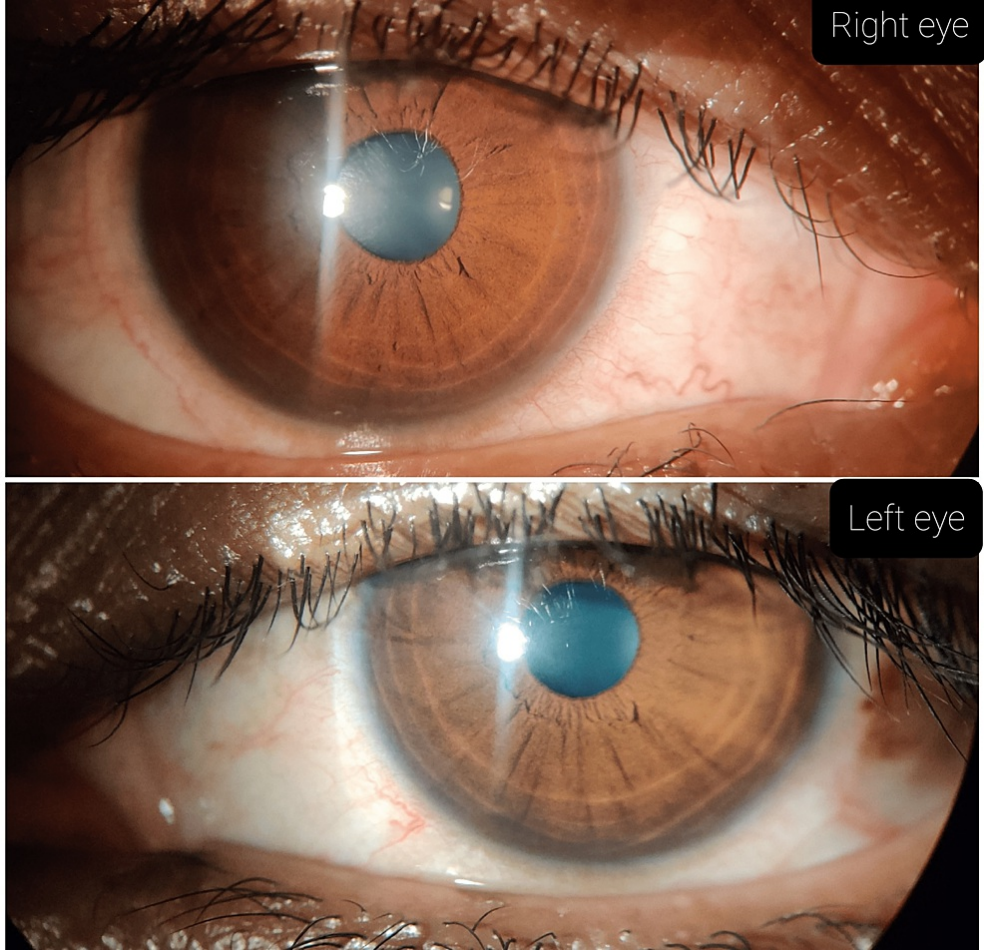
Showing clear cornea in both eyes after three weeks of treatment

The patient was advised to do lid-scrub with baby shampoo and warm water and apply eye ointment azithromycin 1% and eye drop moxifloxacin (0.5%) four times a day and eye drop carboxymethylcellulose (0.5%) six times a day. She was followed up every week for three weeks and showed improvement in blepharitis and punctate corneal erosions (Figure 4). For acneiform eruptions, she was referred to the dermatology department.

## Discussion

This paper describes a patient who developed blepharitis and punctate corneal erosions while on gefitinib for non-small cell carcinoma of the lung with confirmed exon-19 mutation. On evaluation, she was also found to have corneal thinning and reduced corneal sensation.

EGFR is expressed in the basal cells of limbal and conjunctival epithelia. EGF stimulates the proliferation and migration of epithelial cells, resulting in corneal epithelial healing. It is produced by the lacrimal glands when the corneal epithelium is injured. Elevated levels of EGF are present in tears, which help in maintaining normal ocular surface homeostasis [3]. Thus, ocular toxicity related to gefitinib can be explained by the inhibition of the proliferation and migration of conjunctival and limbal epithelial cells [4,5].

Adverse effects of gefitinib at a dose of 250 mg/day on cornea and tear film are dry eyes, superficial punctate keratopathy, and corneal erosions [3]. However, our patient also had corneal thinning and reduced corneal sensation. There was no evidence of dry eye in our patient as suggested by normal tear film function tests.

There are previous case reports in which patients of higher age groups presented with corneal ulceration and thinning within three months of gefitinib therapy [6,7]. This case differs in that a young female on gefitinib for two years presented with only mild ocular symptoms. Other ocular adverse effects of gefitinib at a dose of 250 mg/day are eye pain, visual disturbance, hyperlacrimation, diplopia, trichiasis, conjunctivitis, cataract, anisocoria, and bilateral macular edema [3].

The cutaneous adverse effects of EGFR inhibitors observed are papulopustular eruptions, xerosis, hair growth abnormalities, mucositis, paronychia, and pyogenic granuloma [8]. Skin eruptions are seen in areas with an abundant number of sebaceous glands like the face [9]. In our patient, acneiform eruptions were present on the face and around the eyebrows leading to madarosis.

The patient had shown a good systemic response to gefitinib with no active tumor activity on the positron emission tomography scan. However, she had developed corneal thinning and reduced corneal sensation. Hence, an oncology consultation was done in order to reconsider whether gefitinib was to be continued. Her oncologist had decided to continue gefitinib with strict monthly ophthalmology follow-up.

## Conclusions

Gefitinib is a well-tolerated anticancer drug with fewer systemic side effects. However, its ocular side effects can be sight-threatening if not detected early. Therefore, the oncologist and the ophthalmologist should work in tandem for an optimal visual outcome to prevent ocular complications. Oncologists should send patients on gefitinib therapy for periodic ophthalmologic evaluation so that ocular adverse effects can be detected at an early stage.
